# Cytogenetic data for sixteen ant species from North-eastern Amazonia with phylogenetic insights into three subfamilies

**DOI:** 10.3897/CompCytogen.v14i1.46692

**Published:** 2020-01-22

**Authors:** Hilton Jeferson Alves Cardoso de Aguiar, Luísa Antônia Campos Barros, Linda Inês Silveira, Frédéric Petitclerc, Sandrine Etienne, Jérôme Orivel

**Affiliations:** 1 Universidade Federal do Amapá, Campus Binacional – Oiapoque, BR 156, n°3051 Bairro Universidade, 68980-000, Oiapoque, Amapá, Brazil; 2 Laboratório de Sistemática Molecular Beagle, Universidade Federal de Viçosa, Departamento de Biologia Animal, Av. P. H. Rolfs, s/n, 35570-900, Viçosa, Minas Gerais, Brazil; 3 CNRS, UMR EcoFoG, AgroParisTech, CIRAD, INRA, Université de Guyane, Université des Antilles, Campus Agronomique, BP 316, 97379 Kourou Cedex, France; 4 INRA, UMR EcoFoG, AgroParisTech, CIRAD, CNRS, Université de Guyane, Université des Antilles, Campus Agronomique, BP 316, 97379 Kourou Cedex, France

**Keywords:** Formicidae, karyotype, Neotropical ants, biodiversity

## Abstract

Ants play essential roles in most terrestrial ecosystems and may be considered pests for agriculture and agroforestry. Recent morphological and molecular data have challenged conventional ant phylogeny and the interpretation of karyotypic variations. Existing Neotropical ant cytogenetic data focus on Atlantic rainforest species, and provide evolutionary and taxonomic insight. However, there are data for only 18 Amazonian species. In this study, we describe the karyotypes of 16 ant species belonging to 12 genera and three subfamilies, collected in the Brazilian state of Amapá, and in French Guiana. The karyotypes of six species are described for the first time, including that of the South American genus *Allomerus* Mayr, 1878. The karyotype of *Crematogaster* Lund, 1831 is also described for the first time for the New World. For other species, extant data for geographically distinct populations was compared with our own data, e.g. for the leafcutter ants *Acromyrmex balzani* (Emery, 1890) and *Atta sexdens* (Linnaeus, 1758). The information obtained for the karyotype of *Dolichoderus imitator* Emery, 1894 differs from extant data from the Atlantic forest, thereby highlighting the importance of population cytogenetic approaches. This study also emphasizes the need for good chromosome preparations for studying karyotype structure.

## Introduction

Ants are a diverse group of insects comprising more than 16,000 described species and about 6,000 species yet to be described (Ward 2013), and can represent up to 20% of terrestrial animal biomass in tropical regions (Schultz 2000). Considered good indicators of ecosystem diversity or disturbance (reviewed in Andersen 2018), some ant species play important roles in ecosystems (e.g., seed dispersal, plant protection, predation) whereas other species are considered agricultural pests (Hölldobler and Wilson 1990). However, many ant species belong to cryptic species complexes, making accurate description and the understanding of their biogeographical distribution difficult.

Usually, species identification relies on external morphological traits, but this approach is ineffective in cases where two or more species cannot be morphologically differentiated (Bickford et al. 2007). Complementary biological information can be used in these instances (Schlick-Steiner et al. 2010). Considering recent revisions of higher taxa (Ward et al. 2015, 2016, Sosa-Calvo et al. 2017, 2019), cytogenetics could be used to solve taxonomic issues related to the family Formicidae. Cytogenetics is particularly useful in understanding species evolution and population dynamics because chromosome modifications play a direct role in speciation events and generate heritable variation (King 1993, Aguiar et al. 2017).

More than 800 species of Formicidae have been cytogenetically studied to date (reviewed in Lorite and Palomeque 2010, Mariano et al. 2015, 2019). Cytogenetic research of Neotropical ants has focused on species found in the Atlantic forest biome in Brazil, with few data for other regions and countries. Population studies in ant cytogenetics remain scarce, e.g. *Typhlomyrmex rogenhoferi* Mayr, 1862 (Mariano et al. 2006), *Dinoponera lucida* Emery, 1901 (Mariano et al. 2008), *Pachycondyla* spp. (Mariano et al. 2012), and *Camponotus rufipes* (Fabricius, 1775) (Aguiar et al. 2017). However, cytogenetic data can be used to identify cryptic species, which are common in Formicidae (Seifert 2009). Cytogenetic data have advanced our understanding of biology, reproduction, phylogeny, taxonomy, and evolution, and facilitated investigation of cryptic and threatened species (Lorite and Palomeque 2010).

Cytogenetic data are only available for 18 ant species from the Amazon region, mostly (13 species) from French Guiana (Mariano et al. 2006, 2011, 2012, Santos et al. 2010), with four species from the state of Pará, Brazil (Sposito et al. 2006, Mariano et al. 2006, Santos et al. 2012, Mariano et al. 2015), and one species from Amapá, Brazil (Aguiar et al. 2017). Until now, only data for *T. rogenhoferi*, is available for two locations: Pará, Brazil and French Guiana (Mariano et al. 2006). This species shows an interesting cline variation, which highlights the importance of population assays. In the present study, new data for 16 ant species from the Eastern Amazon are presented using cytogenetic analysis (chromosome number and morphology), with phylogenetic insights into three subfamilies.

## Material and methods

Ant colonies were collected in French Guiana at three locations: *Montagne des Singes*, Kourou (5.07225N, 52.69407W), *Campus Agronomique*, Kourou (5.17312N, 52.65480W), and Sinnamary (5.28482N, 52.91403W). Colonies were collected in Brazil at Oiapoque, state of Amapá (3.84151N, 51.84112W) (Table 1). Sampling permission was given by the Instituto Chico Mendes de Conservação da Biodiversidade (ICMBio) to Luísa Antônia Campos Barros (SISBIO accession number 32459). Specimens were identified by Jacques Hubert Charles Delabie and deposited in the reference collection at the Laboratório de Mirmecologia, Centro de Pesquisas do Cacau (CPDC/Brazil), as items #5802 and #5803.

**Table 1.** Ant species cytogenetically studied from North-eastern Amazonia. Diploid (2n) and haploid (n) chromosome numbers, karyotypic formulae, sample sizes (numbers of colonies/individuals) and localities are given.

Species	2n(n)	Karyotypic formula	Col/Ind	Locality
**Subfamily Ponerinae**				
*Anochetus targionii* Emery, 1894*	30	2n = 16m+2sm+2st+10a	1/5	Campus Agronomique, Kourou, FG
*Odontomachus haematodus* Linnaeus, 1758	44	2n = 8sm+18st+18a	3/8	Campus Agronomique, Kourou, FG
*Pseudoponera stigma* Fabricius, 1804	14	2n = 14m	1/4	Oiapoque, BR
*Pseudoponera gilberti* (Kempf, 1960)	12	2n = 10m+2sm	1/6	Sinnamary, FG
**Subfamily Myrmicinae**				
*Atta sexdens* Linnaeus, 1758	22	2n = 18m+2sm+2st	2/12	Campus Agronomique, Kourou, FG; Oiapoque, BR
*Acromyrmex balzani* Emery, 1890	38	2n = 12m+10sm+14st+2a	1/10	Campus Agronomique, Kourou, FG
*Cyphomyrmex transversus* Emery, 1894	24(12)	2n = 14m+6sm+4a (n = 7m+3sm+2a)	2/8	Campus Agronomique, Kourou, FG
*Myrmicocrypta* sp.	30	2n = 22m+2sm+6a	1/6	Sinnamary, FG
*Allomerus decemarticulatus* Mayr, 1878* ; *Hirtella physophora* Martius et Zuccarini, 1832 †	28	2n = 18m+6sm+2a	4/9	La Montagne des Singes, Kourou, FG
*Allomerus octoarticulatus* var. demerarae Mayr, 1878* ; *Cordia nodosa* Lamarck, 1792 †	44	2n = 4sm+40a	5/12	La Montagne des Singes, Kourou, FG
*Allomerus octoarticulatus* Mayr, 1878*; *Hirtella physophora* †	44	2n = 4sm+40a	5/11	La Montagne des Singes, Kourou FG
*Crematogaster longispina* Emery, 1890*	24	2n = 20m+4sm	1/4	Sinnamary, FG
*Strumigenys diabola* Bolton, 2000*	40	2n = 18sm+12st+10a	1/3	Sinnamary, FG
*Wasmannia auropunctata* Roger, 1863	32	2n = 16m+13sm+5st	1/6	Campus Agronomique, Kourou, FG
*Solenopsis geminata* Fabricius, 1804	32 (16)	2n = 14m+12sm+6st (n = 7m+6sm+3st)	1/5	Sinnamary, FG
**Subfamily Dolichoderinae**				
*Dolichoderus imitator* Emery, 1894	46	2n = 6m+28sm+12a	1/5	Sinnamary, FG

* first cytogenetic report. † host plant. FG: French Guiana, BR: Brazil

Metaphases were obtained from the cerebral ganglia of the larvae after meconium elimination, according to Imai et al. (1988). Chromosome number and morphology of metaphases were analyzed using conventional 4% Giemsa staining. Chromosome morphology was defined according to Levan et al. (1964) using the ratio of chromosome arms (long arm/short arm). Metaphases and chromosomes were karyotyped using Adobe Photoshop CC and measured using Image Pro Plus.

## Results and discussion

Sixteen ant species belonging to 12 genera and three subfamilies have been cytogenetically analyzed (Table 1). The karyotypes of six species are described for the first time, including karyotypic information for the genus *Allomerus* Mayr, 1878. Another genus, *Crematogaster* Lund, 1831, is cytogenetically analyzed for the first time in the Neotropical region. Karyotypes of ten species, including the leafcutter ants *Acromyrmex* Mayr, 1865 and *Atta* Fabricius, 1804, previously described in other localities, were compared with our own data.

### Ponerinae: Ponerini: *Anochetus* and *Odontomachus*

*Anochetus* Mayr, 1861 is a monophyletic genus and a sister genus of *Odontomachus* Latreille, 1804 (Larabee et al. 2016, Fernandes 2017). Morphologically, they belong to the subtribe Odontomachiti of trap-jaw ants (Brown-Jr 1976).

**Figure 1.**
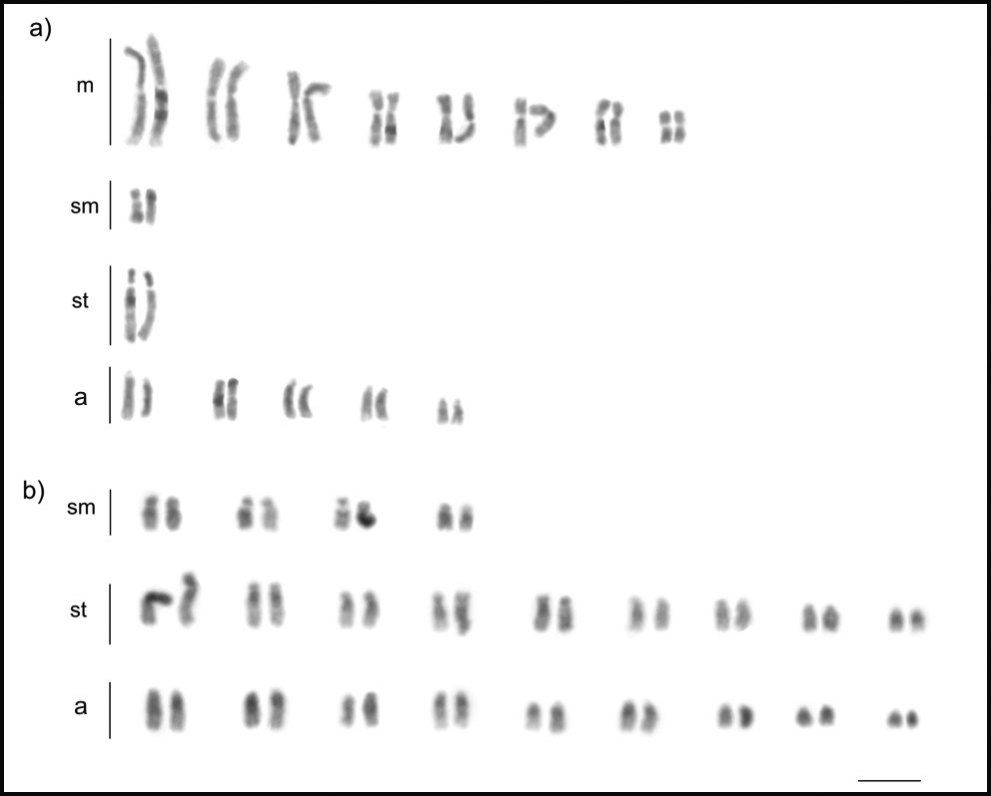
Karyotypes of the tribe Odontomachiti (Ponerinae): **a***Anochetus targionii* (2n = 30) **b***Odontomachus haematodus* (2n = 44). Scale bar: 5µm.

*Anochetus targionii* has 2n = 30 chromosomes (Fig. 1a), which is considered as a modal number according to Santos et al. (2010). *Anochetus* chromosome numbers range from 2n = 24–46, which represents higher karyotype diversity than that found in *Odontomachus* (2n = 32–42) (reviewed in Mariano et al. 2019). However, only 12 morphospecies out of 113 valid species of *Anochetus* have been cytogenetically analyzed: nine from the Indo-Malayan and three from the Neotropics, *A. altisquamis* Mayr, 1887 (2n = 30), *A. horridus* Kempf, 1964 (2n = 46), and *A. emarginatus* (Fabricius, 1804) (2n = 28) (Santos et al. 2010, Mariano et al. 2015).

Since *Anochetus* diversified earlier than *Odontomachus* (Larabee et al. 2016, Fernandes 2017), higher karyotypic variation in *Anochetus* would be expected (Santos et al. 2010). *Anochetus targionii* has the same chromosome number as *A. altisquamis*, *A. modicus* Brown, 1978, and *A. graeffei* Mayr, 1870. It seems that 2n = 30 chromosomes is the plesiomorphic condition since it is found throughout the genus *Anochetus* and is present in *A. altisquamis*, which is considered a phylogenetically “basal” clade (Larabee et al. 2016, Fernandes 2017). *Anochetus* species also share a constant number of acrocentric chromosomes. Within the lineage of *A. horridus*, chromosome fission seems to have played an important role in recent karyotype evolution, increasing the number of chromosomes in the karyotype. According to Larabee et al. (2016), *A. horridus* diversified around 25 million years ago (MYA), whereas *A. targionii* diversified less than 10 MYA.

*Odontomachus haematodus* has 2n = 44 chromosomes, of which 18 are acrocentric (Fig. 1b), confirming information provided by Santos et al. (2007, unpublished data) and Aguiar et al. (2012, unpublished data) (reviewed in Mariano et al. 2019). Similar to *Anochetus*, *Odontomachus* species seem to have characteristic karyotypes that are slightly different between *Odontomachus* phylogenetic clades.

The Indo-Pacific species, *O. rixosus* Smith, 1857 and *O. latidens* Mayr, 1867, have 2n = 30 chromosomes but no further information about their karyotypes is available (reviewed in Lorite and Palomeque 2010). The other known karyotype of *Odontomachus* species belongs to the *haematodus* group according to molecular phylogeny (Larabee et al. 2016). All known karyotypes from the *haematodus* group (reviewed in Santos et al. 2010) have 44 chromosomes, including *O. haematodus*, whose karyotype is described in this study. *Odontomachus chelifer* (Latreille, 1802) has plesiomorphic traits and the highest number of acrocentric chromosomes (40) among Indo-Pacific species (reviewed in Santos et al. 2010). The species *O. meinerti* Forel, 1905 and *O. bauri* Emery, 1892 have 34 and 14 acrocentric chromosomes out of 44 respectively (Teixeira 2018).

This suggests that heterochromatin growth at telomeric regions of shorter arms of acrocentric chromosomes may be significant in *Odontomachus* karyotype evolution. This is in accordance with the Minimum Interaction Theory proposed by Imai et al. (1994), which proposes that reduced interactions between different chromosomes inside the nucleus increases the fitness of the individual.

### Ponerinae: Ponerini: *Pseudoponera*

The genus *Pseudoponera* Emery, 1900 has six valid species (Bolton 2019). Two species *Pseudoponera gilberti* and *P. stigma* are near-identical morphologically. Conflicting cytogenetic analyses (Mariano et al. 2012) due to misidentification have recently been resolved and the two species distinguished in samples from the Atlantic forest (Correia et al. 2016). The chromosome number for *P. gilberti* is 2n = 12 (10m + 2sm) and for *P. stigma* was 2n = 14, all of them metacentrics. In spite of minor differences in chromosome morphology of *P. stigma* between Atlantic forest (Correia et al. 2016) and Amazonia, both karyotypes share the same chromosome number.

**Figure 2.**
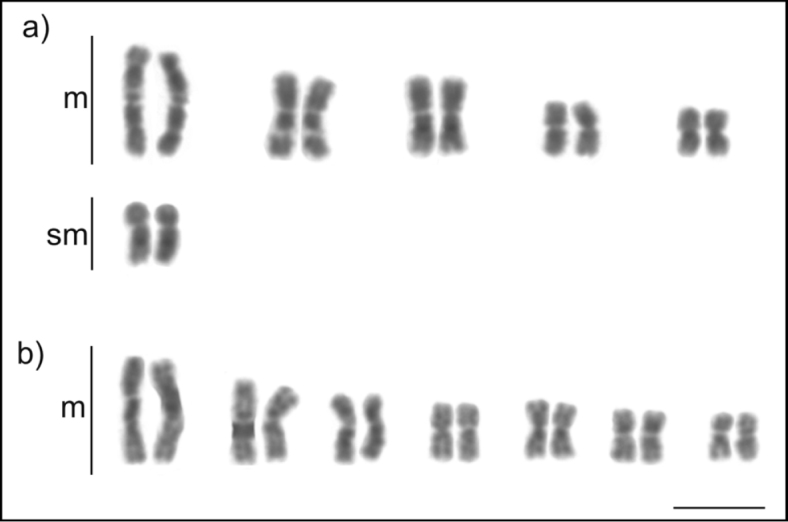
Karyotypes of the genus *Pseudoponera* (Ponerinae): **a***P. gilberti* (2n = 12) **b***P. stigma* (2n = 14). Scale bar: 5µm.

### Myrmicinae: Attini: Attina: *Atta* and *Acromyrmex*

The fungus-growing ants from the genus *Acromyrmex* form a sister group of the genus *Atta* and together are believed to be monophyletic. There are 33 valid species of *Acromyrmex* and 18 species of *Atta* (Bolton 2019), with wide distributions throughout the Neotropics (Delabie et al. 2011). The relationship between *Atta* and *Acromyrmex* became clearer under a combined approach using morphological, molecular, and cytogenetic tools (Cristiano et al. 2013). Cytogenetic data are available for five *Atta* species (Barros et al. 2011, 2014, 2015) from three of the four monophyletic groups according to the molecular phylogeny proposed by Bacci et al. (2009) and 13 species of *Acromyrmex* (reviewed in Barros et al. 2011, 2016, Teixeira et al. 2017).

**Figure 3.**
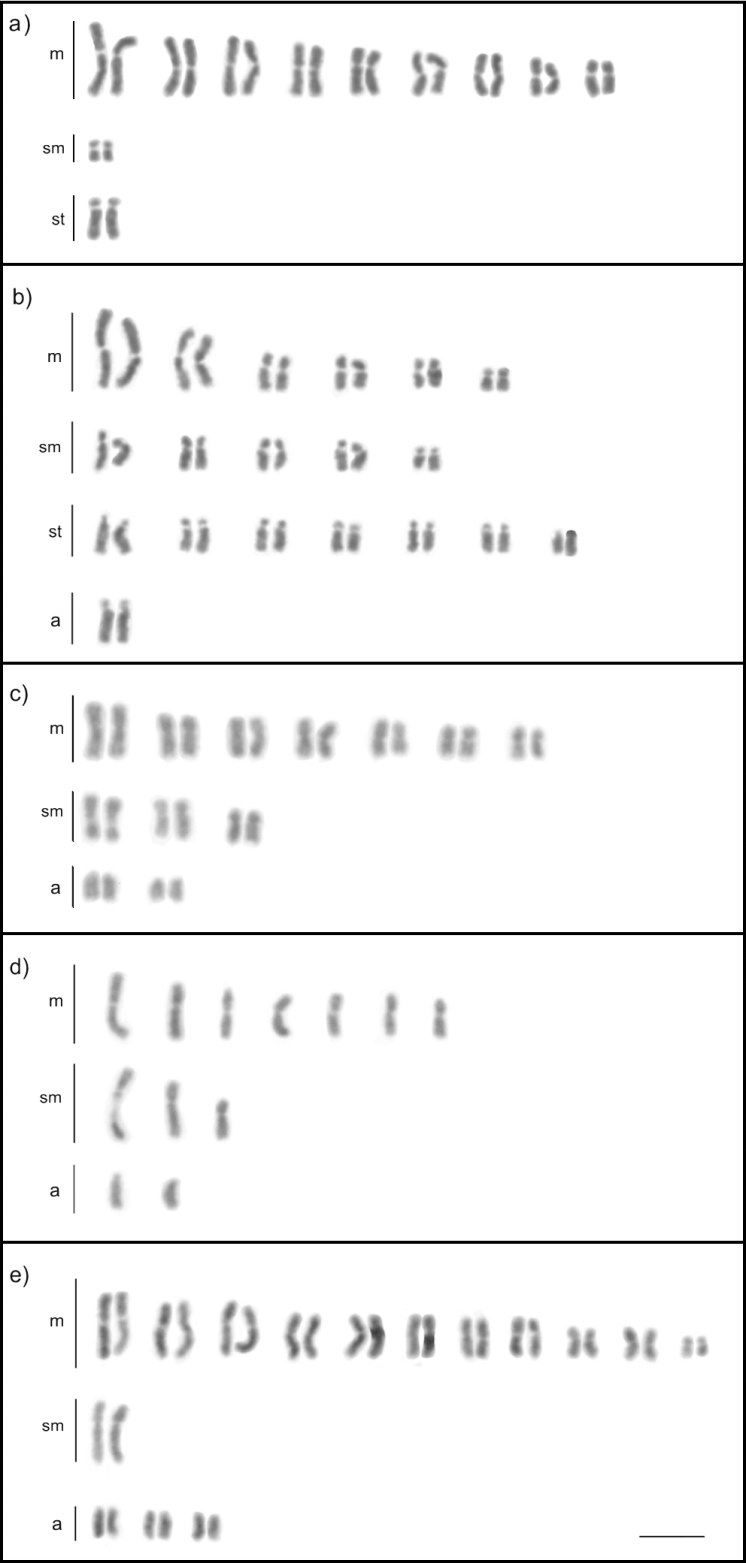
Karyotypes of fungus-growing ants (Myrmicinae, Attini: Attina): **a***Atta sexdens* (2n = 22) **b***Acromyrmex balzani* (2n = 38) **c***Cyphomyrmex transversus* (2n = 24) **d***C. transversus* (n = 12, male karyotype) **e***Myrmicocrypta* sp. (2n = 30). Scale bar: 5µm.

The leaf-cutter ant *Atta sexdens* has 2n = 22 (Fig. 3a), and chromosome morphology is the same (18m + 2sm + 2st) to that of other *Atta* species from the Brazilian savannah and Atlantic Forest (Barros et al. 2014, 2015). The Amazonian population of *Acromyrmex balzani* analyzed in this study has 2n = 38 chromosomes and the same karyotype (Fig. 3b) as that of the Brazilian savannah and Atlantic forest populations (Barros et al. 2016). The largest metacentric pair of *A. balzani* is large, about twice the length of the largest subtelocentric chromosome previously identified in other Brazilian populations of this species (Barros et al. 2016). In all other *Acromyrmex* species studied so far, the former pair of chromosomes is of similar length. Based on the recent checklist of the ants of French Guiana (Franco et al. 2019) this is the first record for *A. balzani* in French Guiana and also the first cytogenetic analysis of this species in the region.

So far, all karyotype analyses showed that *Atta* spp. have 2n = 22 chromosomes and *Acromyrmex* spp. have 2n = 38 chromosomes (Barros et al. 2016, Teixeira et al. 2017). *Acromyrmex striatus* (Roger, 1863) is an exception, with 2n = 22 chromosomes, the same as *Atta* spp. and is considered the sister group of leaf-cutter ants (Cristiano et al. 2013). There are variations between the morphological features of certain chromosomes in *Acromyrmex* due to heterochromatin growth (Barros et al. 2016). Interpopulation cytogenetic studies for ants are scarce (e.g., Mariano et al., 2006, 2012, Aguiar et al. 2017) and none are available for leaf-cutter ants.

### Myrmicinae: Attini: Attina: *Cyphomyrmex*

The fungus-growing attine *Cyphomyrmex transversus* has 2n = 24 and n = 12 with mostly metacentric and submetacentric chromosomes (Fig. 3c, d) which differs from 2n = 42, all of them acrocentric, observed by Mariano et al. (2019), highlighting the importance of detailed cytogenetic studies in this species. It has a range from northern Brazil to central Argentina including the northeastern regions of Brazil (Kempf 1965). *Cyphomyrmex transversus* and the three other *Cyphomyrmex* species which have been karyotyped (see Sosa-Calvo et al. 2017 for recent taxonomic changes) have chromosome numbers ranging between 2n = 20 and 2n = 42 (reviewed in Mariano et al. 2019). It seems that the high proportion of metacentric chromosomes is characteristic of this genus. In spite of morphological affinity of *C. transversus* (present study) to *C. rimosus* (Spinola, 1851), observed by Kempf (1965), the karyotype divergence between them (2n = 24 and 2n = 32, respectively; Murakami et al. 1998) is puzzling because of the numerical difference of their karyotypes coupled with similar morphology of chromosomes. These findings merit further study using advanced chromosome banding and molecular phylogenetic techniques.

### Myrmicinae: Attini: Attina: *Myrmicocrypta*

The fungus-growing species *Myrmicocrypta* sp. had 2n = 30 chromosomes, 18 of them metacentric (Fig. 3e). The studied colony was collected from the cavities of a rotten log. *Myrmicocrypta* Smith, 1860 is the sister genus of *Mycocepurus* Forel, 1893, and both are members of the clade Palleoattina (Sosa-Calvo et al. 2017). *Myrmicocrypta* is widely distributed in the Neotropics, from Mexico to Argentina and includes 27 valid species (Bolton 2019). A recent study by Sosa-Calvo et al. (2019) suggests that only two species can nest in rotten logs: *M. spinosa* Weber, 1937 and the undescribed species, *M.* JSC001. This is a derived characteristic for *Myrmicocrypta* and therefore this clade is apparently monophyletic. The only extant cytogenetic data available for this genus are from the *Montagne des Singes* area, French Guiana (Mariano et al. 2011), about 60 km from where the samples from the present study were collected.

Since the studied sample was identified as an undescribed species, it is possible that the present species is *M.* JSC001. *Myrmicocrypta spinosa* has not been recorded in French Guiana: the samples studied by Sosa-Calvo et al. (2019) included only *M.* JSC001. The species studied by Mariano et al. (2011) had a slightly different karyotype, probably as a result of variation in the chromosome condensation. These results highlight the importance of good chromosome preparations for studying karyotype configuration.

### Myrmicinae: Attini: *Allomerus*

**Figure 4.**
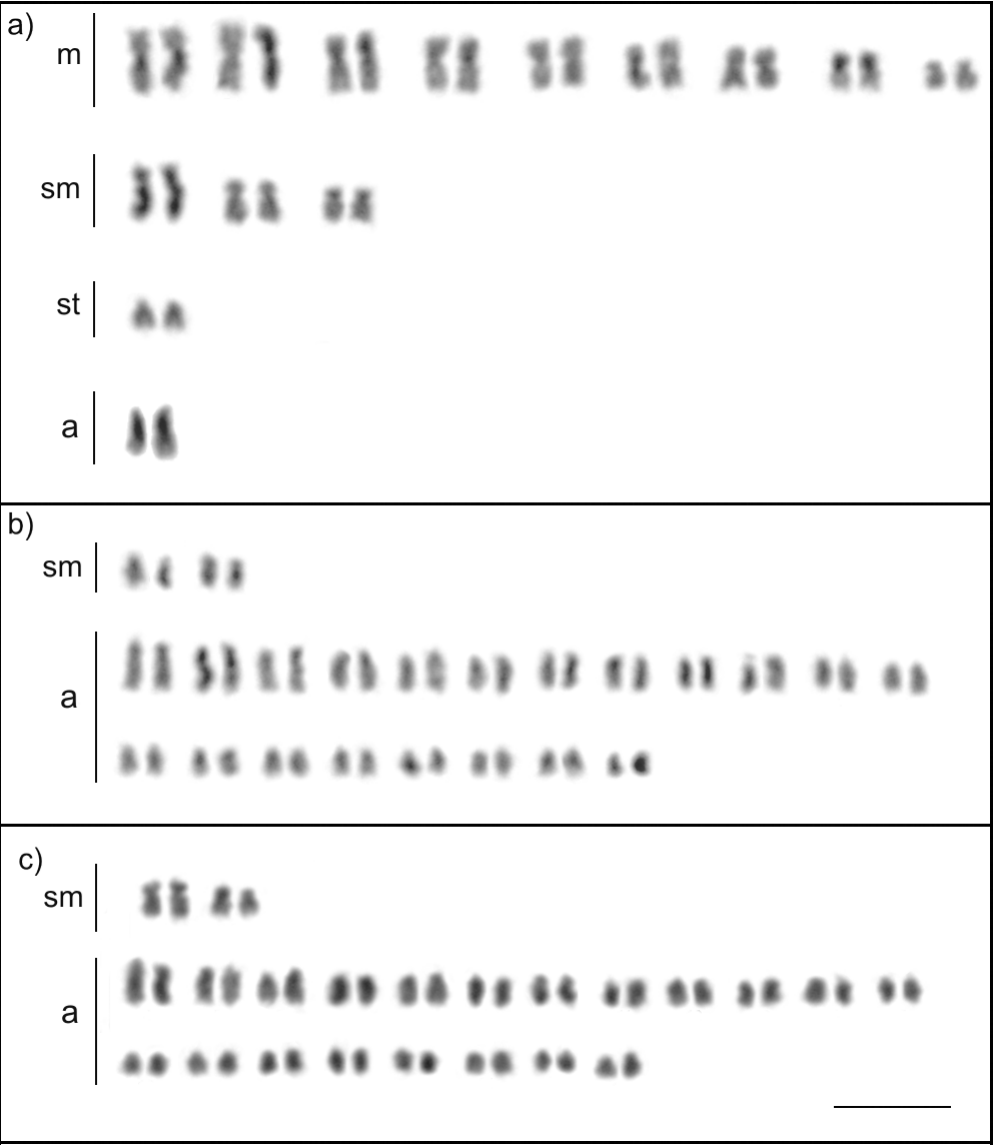
Karyotypes of the genus *Allomerus* (Myrmicinae): **a***A. decemarticulatus* (2n = 28) **b**A. octoarticulatus var. demerarae (2n = 44) associated with *Cordia nodosa***c***A. octoarticulatus* (2n = 44) associated with *Hirtella* sp. Scale bar: 5µm.

This study represents the first cytogenetic analysis for the genus *Allomerus*. *A. decemarticulatus* and the *A. octoarticulatus* species complex had 2n = 28 and 2n = 44, respectively (Fig. 4). The *Allomerus* species are specialist ants inhabiting diverse obligate myrmecophytic plants in South America. *Allomerus decemarticulatus* and *A. octoarticulatus*, have been intensively studied in French Guiana from an ecological perspective: they build galleries using the fungus *Trimmatostroma cordae* Sharma & Singh, 1976 (see Dejean et al. 2005, Ruiz-González et al. 2011). The molecular phylogeny of the genus showed that *A. octoarticulatus* is a complex of two species that cannot be separated morphologically (Orivel et al. 2017). However, these two species are associated with different plants: A. octoarticulatus var. demerarae inhabits only *Cordia nodosa*, while *A. octoarticulatus* can be associated with several myrmecophytic plant species throughout its distribution range.

The number of acrocentric chromosomes is highly different between these two species, even though meta/submetacentric and acrocentric chromosomes predominate in *A. decemarticulatus* and *A. octoarticulatus*, respectively. According to the Minimum Interaction Theory, centric fissions may have played an important role in the chromosome evolution of *Allomerus*; however, the karyotypes of additional species should be investigated to support this conclusion. A comparison between the two species of *A. octoarticulatus*, which nest in different plant species, was also made (Fig. 4b, c). However, basic cytogenetic techniques (chromosome number and morphology) could not differentiate between the two. Additional banding techniques with molecular probes may further illuminate this question in the future.

### Myrmicinae: Attini: *Strumigenys*

**Figure 5.**
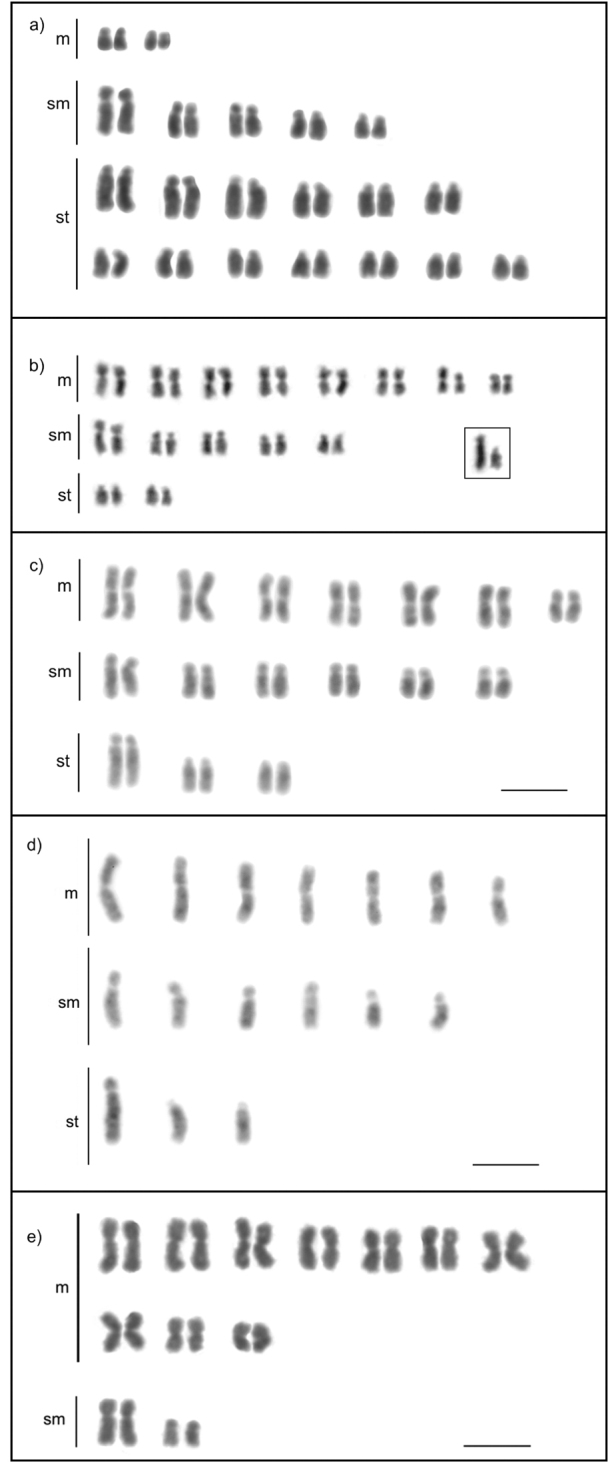
Karyotypes of four genera of Myrmicinae: **a***Strumigenys diabola* (2n = 40) **b***Wasmannia auropunctata* (2n = 32) **c***Solenopsis geminata* (2n = 32) **d***S. geminata* (n = 16, male karyotype) **e***Crematogaster longispina* (2n = 24). Box shows the chromosome pair with size heteromorphism. Scale bar: 5µm.

*Strumigenys diabola* has 2n = 40 (Fig. 5a) with many chromosomes having short arms (submeta/subtelocentrics). The genus *Strumigenys* Smith, 1860 harbors small cryptic species specialized in preying on collembolans. There are currently more than 800 valid species of *Strumigenys* (Bolton 2019) of which 190 are from the Neotropics. *Strumigenys diabola* are reported in northern and northeastern Brazil and in French Guiana (Janicki et al. 2016). For the Neotropics, cytogenetic data was previously only available for *Strumigenys louisianae* Roger, 1863, which has 2n = 4 (Alves-Silva et al. 2014) and for eight species from Asia and Oceania (Lorite and Palomeque 2010). This is the second cytogenetic record in Neotropics and the absence of data in the *Strumigenys mandibularis* group sensu Bolton (2000) make comparisons with other species impossible. Further studies of this genus will help understanding chromosome evolution and phylogeny of the group.

### Myrmicinae: Attini: *Wasmannia*

In this study, workers of *W. auropunctata* had 2n = 32 (Fig. 5b), with one chromosome pair showing considerable size heteromorphism in all individuals analyzed. The genus *Wasmannia* Forel, 1893 includes 11 species and is endemic to the Neotropics. The “little fire ant” *W. auropunctata* is notable because of its reproductive mechanism (Fournier et al. 2005). It has three different genetic systems: haplodiploidy, male clonality, and thelytoky (Foucaud et al. 2006). In this study, the same chromosome number and a similar karyotype from colonies from Ilhéus and Una, southeast Bahia, Brazil were observed (Souza et al. 2011), although there are differences in chromosome classification. The heteromorphic pattern was not described for the Atlantic forest population and therefore needs to be investigated further.

### Myrmicinae: Solenopsidini: *Solenopsis*

Our analysis found 2n = 32 in female *Solenopsis geminata* and n = 16 in males with most chromosomes (26) being metacentric or submetacentric (Fig. 5c, d). The genus *Solenopsis* Westwood, 1840 is difficult to identify at the species level, although these species form obvious natural groups (Pacheco and Mackay 2013). The chromosome number for this species in our analysis is the same as that observed in five previously described fire ant species including *S. geminata*, (reviewed in Lorite and Palomeque 2010) which belong to the subgenus Solenopsis (Pacheco and Mackay 2013).

We compared our data with those from colonies of *S. geminata* from the USA (Crozier 1970) and India (Imai et al. 1984). The karyotype from French Guiana is similar to that from India, despite certain differences in chromosome classification. Differences in karyotypic formula among various localities and colonies were reported by Imai et al. (1984) based on their observation of the presence/absence of the short arm in some chromosomes as a result of C-band polymorphisms. Those patterns demonstrate the importance of understanding heterochromatin dynamics at the population level for analyzing karyotype evolution of ants.

### Myrmicinae: Crematogastrini: *Crematogaster*

The ant genus *Crematogaster* is a global, widespread, and species-rich clade. It currently comprises 498 valid species and is divided into two subgenera, C*rematogaster* sensu stricto and C*rematogaster* (*Orthocrema*) Santschi, 1918 (Blaimer 2012a, b). The subgenus Orthocrema is more complex, and numerous clades exist within this group. *Crematogaster longispina*, which belongs to the subgenus Orthocrema, has 2n = 24, and all chromosomes are meta/submetacentrics (Fig. 5e). This is the first New World *Crematogaster* karyotype ever described, which makes reasonable comparisons difficult. Karyotype data is available for 17 morphospecies of *Crematogaster* from Malaysia, Indonesia, India, Japan, and Australia (reviewed in Lorite and Palomeque 2010).

Within *Crematogaster* spp., the chromosome number ranges from 2n = 24–58, with 10 morphospecies having 2n = 24 or 26. Increasing the number of studied species in the Neotropics may help to understand the chromosome evolution of the group.

### Dolichoderinae: *Dolichoderus*

*Dolichoderus* Lund, 1831 is the most speciose ant genus in the subfamily *Dolichoderinae*, with 130 valid species (Bolton 2019). Chromosomal data is available for seven species collected from the Atlantic forest (Santos et al. 2016) and four species from the Indo-Malayan region (reviewed in Lorite and Palomeque 2010). The genus demonstrates high chromosome variation, 2n = 10–52, and is the most cytogenetically diverse genus within Dolichoderinae. According to the molecular phylogeny produced by Santos et al. (2016), this species occupies a less derived position, which agrees with previous conclusions that suggest that this species belongs to a separate species group (Mackay 1993). The chromosome number already known for this species is 2n = 38, and it also has many meta/submetacentric chromosomes (Santos et al. 2016). However, in this study, additional acrocentric chromosomes were observed in *D. imitator* (2n = 46). Chromosomal intraspecific variation in *Dolichoderus* has not previously been reported. This again emphasizes the importance of karyotypic studies at the level of certain populations, which may represent either geographic clines or a species complex. Enhancing population studies for this species may have important implications for our understanding of both taxonomy and chromosome evolution of Formicidae.

**Figure 6.**
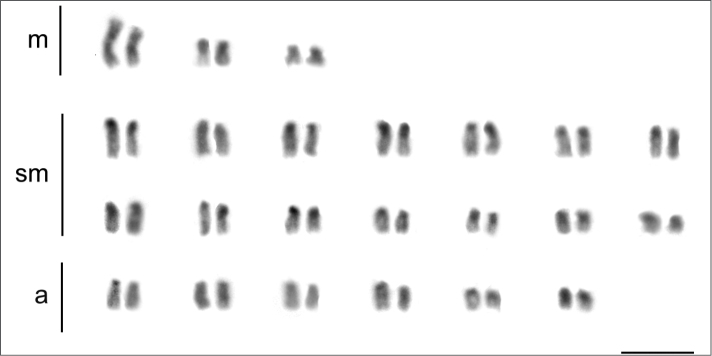
Karyotype of *Dolichoderus imitator* (2n = 46) (Dolichoderinae). Scale bar: 5µm.

## Conclusion

Our study increased the number of karyotyped Amazonian ant species from 18 to 34. The karyotype of 16 species were analyzed, six of them for the first time, which permitted comparisons with previously studied species, including population studies of leaf-cutting ants (*Atta sexdens* and *Acromyrmex balzani*). Although cytogenetic analysis of more than 800 ant species is available, there are no data for many genera, including many Neotropical ones. This paper includes the first description of the karyotype of a *Crematogaster* species ever reported for the New World.

Conventional cytogenetics constitutes a powerful tool in characterizing cryptic biodiversity (Cioffi et al. 2018). For example, our study of *D. imitator* showed substantial differences between chromosome numbers of the previously studied Atlantic forest karyotype and that of our study, strongly suggesting the presence of different species. Future studies on molecular cytogenetics will have important implications for understanding the chromosome evolution of ants, focusing especially on the genus *Allomerus* and fungus-growing ants.
